# Immune Monitoring in Melanoma and Urothelial Cancer Patients Treated with Anti-PD-1 Immunotherapy and SBRT Discloses Tumor Specific Immune Signatures

**DOI:** 10.3390/cancers13112630

**Published:** 2021-05-27

**Authors:** Annabel Meireson, Simon J. Tavernier, Sofie Van Gassen, Nora Sundahl, Annelies Demeyer, Mathieu Spaas, Vibeke Kruse, Liesbeth Ferdinande, Jo Van Dorpe, Benjamin Hennart, Delphine Allorge, Filomeen Haerynck, Karel Decaestecker, Sylvie Rottey, Yvan Saeys, Piet Ost, Lieve Brochez

**Affiliations:** 1 Cancer Research Institute Ghent (CRIG), Ghent University, 9000 Ghent, Belgium; annabel.meireson@ugent.be (A.M.); nora.sundahl@ugent.be (N.S.); annelies.demeyer@ugent.be (A.D.); mathieu.spaas@ugent.be (M.S.); vibeke.kruse@ugent.be (V.K.); jo.vandorpe@ugent.be (J.V.D.); karel.decaestecker@ugent.be (K.D.); sylvie.rottey@ugent.be (S.R.); yvan.saeys@ugent.be (Y.S.); piet.ost@ugent.be (P.O.); 2 Dermatology Research Unit, Ghent University Hospital, 9000 Ghent, Belgium; 3 Centre for Primary Immunodeficiency Ghent, Primary Immune Deficiency Research Lab, Department of Internal Medicine and Pediatrics, Jeffrey Modell Diagnosis and Research Centre, Ghent University Hospital, 9000 Ghent, Belgium; simon.tavernier@irc.vib-ugent.be (S.J.T.); filomeen.haerynck@ugent.be (F.H.); 4 VIB Center for Inflammation Research, Unit of Molecular Signal Transduction in Inflammation, 9000 Ghent, Belgium; 5 VIB Center for Inflammation Research, Unit of Data Mining and Modeling for Biomedicine, 9000 Ghent, Belgium; sofie.vangassen@ugent.be; 6 Department of Applied Mathematics, Computer Science and Statistics, Ghent University, 9000 Ghent, Belgium; 7 Department of Radiation Oncology and Experimental Cancer Research, Ghent University Hospital, 9000 Ghent, Belgium; 8 Department of Medical Oncology, Ghent University Hospital, 9000 Ghent, Belgium; 9 Department of Pathology, Ghent University Hospital, 9000 Ghent, Belgium; liesbeth.ferdinande@ugent.be; 10 Unité Fonctionnelle de Toxicologie, CHU Lille, F-59000 Lille, France; benjamin.hennart@CHRU-LILLE.FR (B.H.); delphine.allorge@CHRU-LILLE.FR (D.A.); 11 ULR 4483-IMPact de l’Environnement Chimique sur la Santé Humaine (IMPECS), Université de Lille, F-59000 Lille, France; 12 Department of Urology, Ghent University Hospital, 9000 Ghent, Belgium

**Keywords:** immunotherapy, anti-PD-1, melanoma, urothelial cancer, immune monitoring, blood biomarkers

## Abstract

**Simple Summary:**

Currently available biomarkers for response to checkpoint inhibitors are incomplete and predominantly focus on tumor tissue analysis e.g., tumor mutational burden, programmed cell death-ligand 1 (PD-L1) expression. Biomarkers in peripheral blood would allow a more dynamic monitoring and could offer a way for sequential adaptation of treatment strategy. We conducted an in-depth analysis of baseline and on-treatment systemic immune features in a cohort of stage III/IV melanoma and stage IV urothelial cancer (UC) patients treated with anti-programmed cell death-1 (anti-PD-1) therapy combined with stereotactic body radiotherapy (SBRT) in a similar regimen/schedule. Baseline immunity was clearly different between these two cohorts, indicating a less active immune landscape in UC patients. This study also detected signatures of proliferation in the CD8^+^ T-cell compartment pre-treatment and early after anti-PD-1 initiation that were positively correlated with clinical outcome in both tumor types. In addition our data support the biological relevance of PD-1/PD-L1 expression on circulating immune cell subsets, especially in melanoma.

**Abstract:**

(1) Background: Blockade of the PD-1/PD-L1 pathway has revolutionized the oncology field in the last decade. However, the proportion of patients experiencing a durable response is still limited. In the current study, we performed an extensive immune monitoring in patients with stage III/IV melanoma and stage IV UC who received anti-PD-1 immunotherapy with SBRT. (2) Methods: In total 145 blood samples from 38 patients, collected at fixed time points before and during treatment, were phenotyped via high-parameter flow cytometry, luminex assay and UPLC-MS/MS. (3) Results**:** Baseline systemic immunity in melanoma and UC patients was different with a more prominent myeloid compartment and a higher neutrophil to lymphocyte ratio in UC. Proliferation (Ki67^+^) of CD8^+^ T-cells and of the PD-1^+^/PD-L1^+^ CD8^+^ subset at baseline correlated with progression free survival in melanoma. In contrast a higher frequency of PD-1/PD-L1 expressing non-proliferating (Ki67^−^) CD8^+^ and CD4^+^ T-cells before treatment was associated with worse outcome in melanoma. In UC, the expansion of Ki67^+^ CD8^+^ T-cells and of the PD-L1^+^ subset relative to tumor burden correlated with clinical outcome. (4) Conclusion: This study reveals a clearly different immune landscape in melanoma and UC at baseline, which may impact immunotherapy response. Signatures of proliferation in the CD8^+^ T-cell compartment prior to and early after anti-PD-1 initiation were positively correlated with clinical outcome in both cohorts. PD-1/PD-L1 expression on circulating immune cell subsets seems of clinical relevance in the melanoma cohort.

## 1. Introduction

New insights in immuno-oncology and the subsequently developed immunotherapies have caused a major breakthrough in the oncology field in the last decade, creating the hope of curing (metastatic) cancer. Despite the encouraging results, the proportion of patients experiencing a durable response is still limited. In 2018 about 43% of cancer patients in the United States were eligible for checkpoint inhibitor therapy compared to 1.5% in 2011, while the estimated percentage of response only modestly increased from 0.14% to 12.4% in the same time period [1]. Combination strategies are currently being tested in different cancer types in an attempt to improve response rates [2,3], but the combination of cytotoxic lymphocyte antigen 4 (CTLA-4) blockade and programmed cell death receptor 1 (PD-1) blockade is well recognized to inevitably elicit higher toxicity and also implies a higher cost. Both from the patient’s and healthcare budget’s perspective there is a need for new translational insights that could help to optimize current immunotherapies.

Up to date predictive biomarkers have mainly been identified in tumor tissue. The immunohistochemical expression of PD-L1 is currently one of the most widely used biomarkers and, high expression has been correlated with response to PD-1/PD-L1 immunotherapy [4,5]. However, a systematic evaluation of 45 Food and Drug Administration (FDA) approved trials involving 15 tumor types demonstrated that PD-L1 expression was predictive in only 28.9% of cases [6]. High tumor mutational burden is also associated with better response [7,8] and this finding led to FDA approval for checkpoint inhibition in patients with microsatellite instability-high or mismatch repair-deficient solid tumors, irrespective of cancer type [9,10]. Patients who respond to anti-PD-1 therapy exhibit a tumor micro-environment that is enriched for interferon γ (IFNγ) and tumor infiltrating lymphocytes (TILs), the so called ‘hot’ tumors [11,12,13].

Blood-based biomarkers have been far less reported and have not yet entered clinical practice, although they could have the benefit of a dynamic monitoring during the treatment course with the possibility to adapt immunotherapeutic strategies.

In the current study, we performed immune monitoring in patients with inoperable stage III/IV melanoma and patients with stage IV UC who received anti-PD-1 immunotherapy combined with SBRT. The immune landscape before and during treatment was compared between tumor types and the relation to clinical outcome was investigated.

## 2. Materials and Methods

### 2.1. Patient Samples

**Figure 1:**
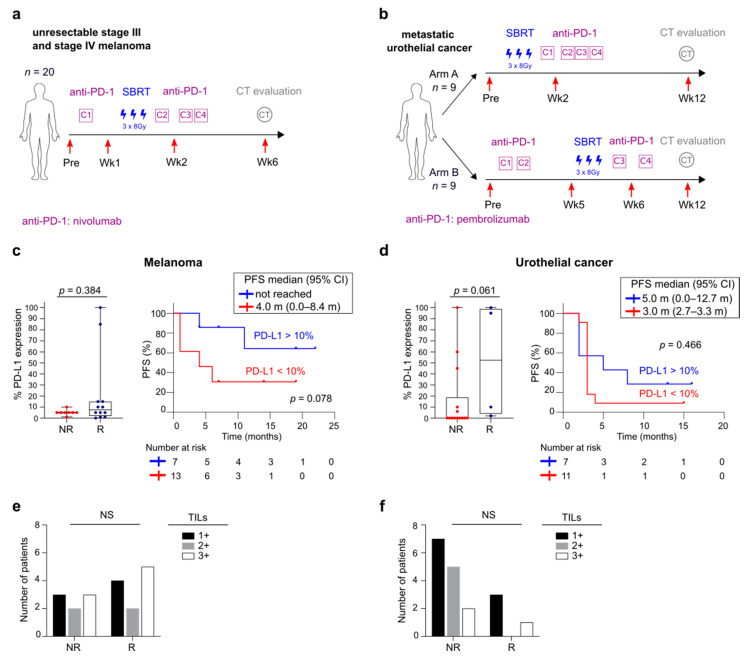
Overview of the clinical trial treatment strategy and PD-L1 and TIL quantification. (**a**) Schematic of design of phase 2 clinical trial in unresectable stage III and stage IV melanoma receiving a combination of anti-PD-1 and SBRT. (**b**) Schematic of design of randomized phase 1 trial combining anti-PD-1 with either sequential (Arm A) or concomitant SBRT (Arm B) in metastatic UC. Red arrows indicate time of blood collection. (**c**) Boxplots with tumoral PD-L1 expression in non-responders and responders (left) and Kaplan-Meier estimate of PFS stratified according to tumoral PD-L1 expression (right) in melanoma and (**d**) in UC. Whiskers of boxplots extend to the minimum and maximum data point, with the horizontal line indicating the median. *p* value calculated using two-sided Mann-Whitney U test (left) and log-rank test (right). (**e**) TIL quantification in non-responders and responders in melanoma and (**f**) in UC. TILs were evaluated semi quantitatively: 1+, sporadic TILs; 2+, moderate number of TILs; 3+, abundant occurrence of TILs. *p* value calculated using Fisher’s exact test. Pre, pre-treatment; Wk, week; Gy, gray; SBRT, stereotactic body radiotherapy; CT, computed tomography; NR, non-responder; R, responder; PFS, progression free survival; NS, not significant; TIL, tumor infiltrating lymphocytes.

The biospecimens evaluated in this study were obtained from patients with melanoma or UC who participated in two separate clinical trials (Figure 1a,b). A phase 2 trial included 20 patients with unresectable stage III or stage IV metastatic melanoma who were treated in the first line with anti-PD-1 (nivolumab) and SBRT (NCT02821182) [14]. The samples from metastatic UC patients were collected during a randomized phase I trial with SBRT administered either prior to the first anti-PD-1 cycle (arm A: SBRT prior to any treatment with pembrolizumab, *n* = 9), or during anti-PD-1 treatment (arm B: SBRT prior to the third pembrolizumab cycle, *n* = 9) [15]. Both trials were approved by the Ethics Committee of Ghent University Hospital and are registered on Clinicaltrials.gov (resp. NCT02821182 and NCT02826564). At fixed time points through treatment, peripheral blood samples (EDTA and serum tubes) were collected from melanoma (*n* = 85) and UC patients (*n* = 60) respectively. Peripheral blood mononuclear cells (PBMCs) were isolated via Lymphoprep centrifugation and stored in liquid nitrogen using standard methods.

Tumor burden was assessed using CT/MRI or PET-CT scan of the chest, abdomen and pelvis at baseline, after the fourth cycle of anti-PD-1 and after every fifth cycle (melanoma) or third cycle (UC) thereafter until the end of treatment. Tumor burden was defined as the sum of the longest diameters for a maximum of five target lesions and up to two lesions per organ. For lymph nodes the shortest axis was measured. Clinical responses were determined based on Response Evaluation Criteria in Solid Tumors (RECIST) 1.1 criteria. Disease control was achieved in 12 melanoma patients (complete response, CR (*n* = 3); partial response; PR (*n* = 6) and stable disease, SD (*n* = 3)) while 8 patients showed progressive disease (non-responder). In UC, no objective responders were observed in arm A, while of 4 patients in arm B achieved a complete or partial response (CR: *n* = 1; PR: *n* = 3).

### 2.2. Flow Cytometry

Cryopreserved PBMCs were thawed and washed in RPMI 1640 medium supplemented with Glutamax (2.05 mM), 10% FCS and penicillin (100 U/mL)-streptomycin (100 µg/mL). Cells were stained with monoclonal antibodies labeled with fluorochromes. A complete list of the used antibodies can be found in Table S3. In a first step, 2.5 × 10^6^ cells were stained with FcR blocking reagent for blocking of unspecific binding of antibodies (130-059-901, Miltenyi, Madrid, Spain) and a mixture of Fixable Viability dye eFluor 506 (65-0866-14, eBioscience, San Diego, CA, USA) and antibodies against surface markers in PBS and BD Horizon Brilliant Stain Buffer (563794, BD Biosciences, San Jose, CA, USA), incubated for 30 min at 4 °C and washed. In a second step, cells were fixed and permeabilized with Foxp3 Transcription Factor Staining Buffer Set (00-5523-00, eBioscience, San Diego, CA, USA), and subsequently stained intracellularly for 30 min at RT. Labeled cell suspensions were acquired on a BD FACSymphony flowcytometer (BD Biosciences, San Jose, CA, USA) and data was analyzed with FlowJo 10.6.2 software (Ashland, OR, USA). Gating strategies are depicted in Figure S1.

The frequency of neutrophils and lymphocytes in white blood cells was determined for all of the samples using automated blood cell counting equipment (Sysmex XE-5000, Norderstedt, Germany) during routine lab evaluations.

### 2.3. High Dimensional Data Analysis of Flow Cytometry Data

#### 2.3.1. t-SNE

Live CD8^+^ T cells were gated in FlowJo v10.6.2 and exported as separate fcs files for melanoma and UC. Populations before and during treatment were randomly down-sampled and subsequently concatenated into 1 file (total events melanoma: 1.234.633 events; total events urothelial cancer arm A: 689.057 events; total events urothelial cancer arm B: 979.821 events). Next, concatenated samples were analyzed via t-distributed stochastic neighborhood embedding (t-SNE) in FlowJo v10.6.2. Opt-SNE was applied as learning configuration, with perplexity set to 30 and iterations to 1000. The colors in the heatmap represent the measured means intensity value of Ki67 in a given cluster.

#### 2.3.2. FlowSOM

The melanoma and UC datasets were analyzed separately, following the same pipeline. The fcs files were first cleaned by manual gating in FlowJo, after which the data was imported in R. An aggregate was generated with approximately 3 million cells, with an equal number of cells subsampled at random without repetition from each sample (melanoma: 85 samples with 35,295 cells each, urothelial cancer arm B: 36 samples with 83,334 cells each). This aggregate was then used to train a FlowSOM model with a 15 by 15 grid (225 clusters) and 30 metaclusters. Thirteen markers were taken along for the clustering: CD3, CD4, CD8, CD25, CD19, CD56, HLA-DR, CD123, CD33, CD11b, CD14, CD16 and FoxP3.

Once the model was built, all samples were fully mapped onto the model, resulting in a cluster and metacluster assignment for each cell. From this mapping, the cluster and metacluster abundances per sample were extracted. Additionally, for 6 markers (CTLA-4, Ki67, IDO, PD-1, PD-L1 and HLA-DR), a positivity threshold was determined by manual gating. We used these thresholds to determine the abundance of each possible subpopulation in each (meta-) cluster. A subpopulation was defined by being either positive, negative or neutral (both positive and negative cells included) for each of the markers, resulting in 729 potential combinations per (meta-) cluster. As many of these combinations would not occur in reality, these subpopulations were then filtered, only keeping those where at least 5 samples had at least 30 cells. This resulted in a total of 76,039 features describing the immune profile of melanoma samples and 70,648 features for the urothelial cancer samples.

### 2.4. Cytokine Measurement

Magnetic luminex assay (R&D systems, Minneapolis, MN, USA) was performed on cryopreserved serum samples according to manufacturer’s instructions using a customized panel, including CXCL9, CXCL10, MICA, MICB, ULBP-1, ULBP-2/5/6, ULBP-3, ULBP-4 and s100B. Serum concentrations were measured on a Bio-Plex 200 Array Reader (Bio-Rad, Hercules, CA, USA).

### 2.5. UPLC-MS/MS

Tryptophan (Trp) and its metabolite kynurenine (Kyn) were quantified according to previously published methods [16,17], with slight modifications. Cryopreserved serum samples (50 µL) were extracted using 50 µL acetonitrile containing Trp-D5 (50 µM, CDN Isotopes, Pointe-Claire, QC, Canada) as an internal standard. The samples were centrifuged (8 min, 11,800 rpm, 4 °C) and the supernatants (50 µL) were added to deionized water (600 µL). Fifteen µL of this mixture was injected in an ultra-high-performance liquid chromatography system coupled to tandem mass spectrometry detector (UPLC-MS/MS, Acquity TQ-S Detector, Waters, Milford, MA, USA) equipped with a HSS C18 column. Ions of each analyzed compound were detected in a positive ion mode using multiple reaction monitoring.

### 2.6. Scoring of PD-L1 and Tumor Infiltrating Cells

Formalin-fixed, paraffin-embedded (FFPE) tumor samples were collected at time of surgical resection before start of systemic treatment in melanoma and UC patients. 4 µm-thick FFPE tissue sections were subjected to heat-induced antigen retrieval and incubated with primary monoclonal antibodies against PD-L1: clone SP263 (Ventana Medical Systems Inc., Tucson, AZ, USA) for melanoma samples and clone 22C3 (Agilent Technologies, Santa Clara, CA, USA) for UC samples. Samples were visualized with 3,3′-diaminobenzidine (DAB) chromogen and hematoxylin counterstain and cover-slipped for review. Scoring of PD-L1 was conducted by 2 pathologists blinded to patient characteristics. In melanoma sections, the percentage of tumor cells with membranous PD-L1 staining was scored (0–100%). In UC sections, the percentage of tumor cells and any tumor infiltrating mononuclear inflammatory cells with membranous PD-L1 staining was scored (0–100%).

The abundance of intraepithelial TILs was determined on H&E stained sections. This morphological assessment of TILs within tumor nests was evaluated semi quantitatively: 1+, sporadic TILs; 2+, moderate number of TILs; 3+, abundant occurrence of TILs. For dichotomization, the TILs score was categorized into ‘low’ (1+ or 2+) and ‘high’ (3+). TILs were assessed on 19 melanoma patients as the only available specimens for the 20th patient was a cytological sample.

### 2.7. Statistics

To compare longitudinal immunologic effects, *p*-values for each measured immune feature were calculated using a Wilcoxon matched-pairs signed-ranks test. Associations between immune features and treatment response were identified by Mann-Whitney U tests comparing the frequencies of phenotypes between responders and non-responders. Progression free survival (PFS) was defined as the time from inclusion to disease progression, death or the last follow-up, whichever occurred first. PFS curves were estimated using the Kaplan-Meier method by dichotomizing immune phenotypes of interest through their median value. Survival curves between patients with high (above the median) and low (below the median) frequencies of the immune feature of interest were compared using a Log-Rank test. Cox regression models have been used to perform univariate analysis. Correlations between continuous variables were determined by Spearman’s r coefficient. A chi square test was employed to test for association between two categorical variables. Fold change in proliferation was calculated by dividing the frequency of Ki67^+^ T-cells in on-treatment samples to the frequency of Ki67^+^ T-cells at pre-treatment. Statistical analyses were performed using IBM SPSS v26 and all tests were performed two-sided; *p* < 0.05 was considered to be statistically significant. Graphs were plotted with Graphpad Prism (GraphPad software Inc., San Diego, CA, USA). For FlowSOM analysis, a Wilcoxon Rank Sum test was executed in R to compare responders and non-responders, after which the features were ranked by *p*-value.

## 3. Results

### 3.1. Overview of Patient Cohorts

Blood samples (*n* = 145) of 20 melanoma patients and 18 UC patients treated with anti-PD-1 therapy combined with SBRT were included (NCT02821182 and NCT02826564). The design of the clinical trials and time points of blood sample collection is schematically presented in Figure 1a,b. The clinical results of these trials have been reported elsewhere [14,15]. Detailed patient characteristics are described in Tables S1 and S2.

The median age in the melanoma cohort was 60.5 years (34.0–80.0 years) and 68.0 years (50.0–84.0 years) in the UC cohort (Mann-Whitney U test, *p* = 0.055). Age was not correlated with clinical outcome in the melanoma cohort (Mann-Whitney U test, *p* = 0.473). In the UC cohort, median age was higher in responders (75.5 years, (71.0–84.0 years)) compared to non-responders (61.0 years (50.0–79.0 years), Mann-Whitney U test, *p* = 0.018). The median tumor burden was lower in melanoma patients compared to UC patients (23.5 mm (10.0–100.0 mm) versus 45.8 mm (12.10–106.90 mm), Mann-Whitney U test, *p* = 0.033). Median baseline tumor burden was not different in responders versus non-responders in the melanoma cohort. Responders in the UC cohort tended to have lower median baseline tumor burden compared to non-responders (arm B: 29.45 mm (12.10–46.90 mm) versus 60.50 mm (44.70–75.00 mm), Mann-Whitney U test, *p* = 0.032, for arm A+B *p* = 0.101). Prior systemic treatment had been administered in 2/20 melanoma patients (anti-CTLA-4 and BRAF-targeted therapy). In the UC cohort, 13/18 patients had been treated with one or more platinum-based chemotherapies prior to enrollment in the study.

Tumoral PD-L1 expression was not significantly related to response or PFS in melanoma or UC patients (Figure 1c,d). No difference in baseline TILs was found between responders and non-responders (Figure 1e,f).

### 3.2. Differences in Baseline Immunity between Melanoma and UC Cohort

**Figure 2:**
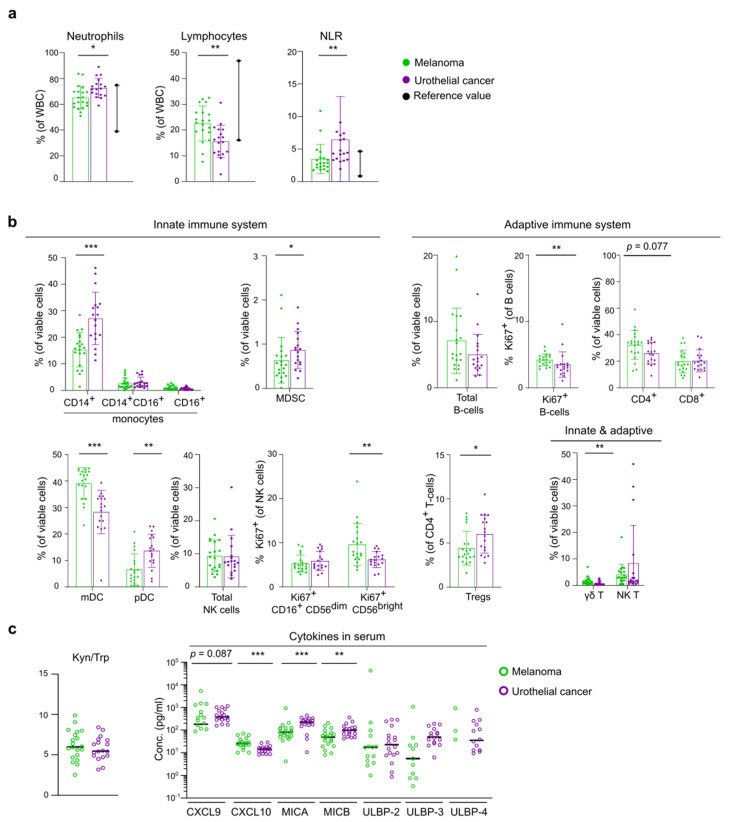
Baseline systemic immunity differs between melanoma and urothelial cancer. (**a**) Frequency of neutrophils, lymphocytes and neutrophil-to-lymphocyte ratio in melanoma and UC. Reference values are depicted in black. (**b**) Frequency of immune cell populations of innate and adaptive immune system. Error bar denotes ± SD. (**c**) (left) Ratio of serum concentrations of kynurenine (Kyn) on tryptophan (Trp), presented values are Kyn/Trp x 100. (right) Serum concentrations of T-cell activating chemokines CXCL9 and CXCL10 and concentrations of ligands for NK cell activing receptor NKG2D: MICA, MICB, ULBP-2, ULBP-3 and ULBP-4. Concentrations out of the range of detection could not be depicted. *p* value calculated using two-sided Mann-Whitney U test. * *p* < 0.05, ** *p* < 0.01, *** *p* < 0.001. WBC, white blood cells; MDSC, myeloid-derived suppressor cells; mDC, myeloid dendritic cells; pDC, plasmacytoid dendritic cells; Tregs, regulatory T-cells; ND, not detectable.

Significant differences in the baseline immune landscape were observed between the melanoma and UC cohort (Figure 2a,b).

The neutrophil-to-lymphocyte ratio (NLR) was higher in UC compared to melanoma. Melanoma patients had a clearly higher lymphocyte frequency and more γδ T-cells and proliferating (Ki67 expressing) B-cells compared to UC patients. There was no significant difference in the frequency of CD4^+^ and CD8^+^ T-cells between the two cohorts, except for the frequency of regulatory T-cells (Tregs, as defined by CD25^+^ Foxp3^+^ CD4^+^ cells [18]) which was lower in melanoma patients.

In the UC cohort, the frequency of neutrophils, classical CD14^+^ monocytes, plasmacytoid dendritic cells (pDCs) and myeloid-derived suppressor cells (MDSCs) was higher compared to the melanoma cohort. Notably, the frequency of CD16^+^ monocytes correlated negatively with tumor burden in UC patients (Spearman’s CC: −0.627, *p* = 0.005). No significant differences were observed in the total NK cell population but the percentage of Ki67^+^ CD56^bright^ NK was lower in the UC cohort.

The baseline concentration of IFNγ-inducible chemokine CXCL10 was higher in melanoma, whereas higher serum concentrations of MICA and MICB-both soluble NKG2D ligands-were detected in UC (Figure 2c). No differences in baseline kynurenine to tryptophan ratio (Kyn/Trp) were observed.

Altogether these data indicate a more favorable baseline immune landscape in the melanoma cohort compared to UC patients.

### 3.3. Early Systemic Immune Changes after Anti-PD-1 Treatment Initiation

To study early dynamic changes in systemic immunity upon anti-PD-1 initiation, blood samples after 1 cycle in the melanoma cohort (collected at week 1) and after 2 cycles in the UC cohort arm B (collected at week 5) were examined.

**Figure 3:**
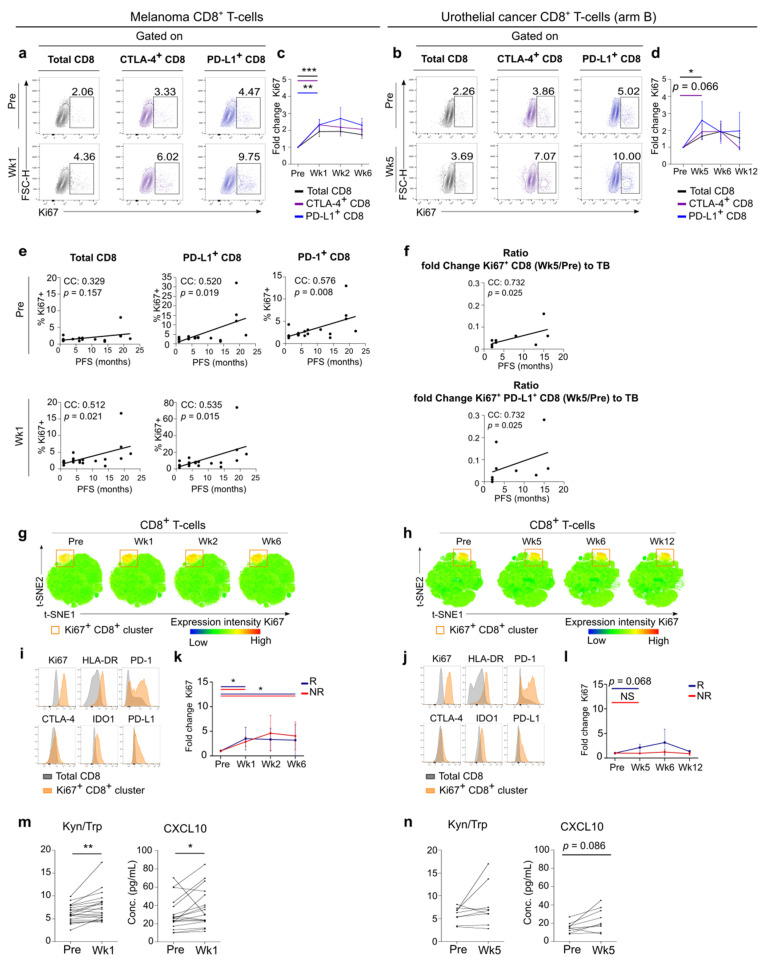
Early upregulation of proliferating CD8^+^ T-cells in response to anti-PD-1. (**a**) Contour plots representing Ki67 expression in CD8^+^ T-cell subsets at pre-treatment (Pre) and after 1 cycle of anti-PD-1 (Week 1) in 12 independent melanoma patients. (**b**) Contour plots representing Ki67 expression in CD8^+^ T-cell subsets at pre-treatment and after 2 cycles of anti-PD-1 (Week 5) in 9 independent UC patients (arm B). (**c**) Lineplot with fold induction of Ki67 expression in CD8^+^ T-cell subsets at indicated times in melanoma (*n* = 20) and (**d**) in UC (arm B, *n* = 9). Data shown are relative to pre-treatment samples. Error bar denotes mean ± SEM. *p* value calculated using two-sided Wilcoxon matched-pairs test. * *p* < 0.05, ** *p* < 0.01. (**e**) Spearman correlation of PFS to Ki67 expression in the total CD8^+^ T-cell population (left), PD-L1^+^ CD8^+^ T-cells (middle) and PD-1^+^ CD8^+^ T-cells (right) at pre-treatment (up) and after 1 cycle of anti-PD-1 (Week 1, down) in melanoma. (**f**) Spearman correlation of PFS to the ratio of the fold change of Ki67 increase on CD8^+^ T-cells (week 5 on pre-treatment) to tumor burden (up) and to the ratio of the fold change of Ki67 increase on PD-L1^+^ CD8^+^ T-cells (week 5 on pre-treatment) to tumor burden (down) in UC. (**g**) *t*-distributed stochastic neighbor embedding (t-SNE) map of CD8^+^ T-cells overlaid with the expression level of Ki67 as a heat map in melanoma and (**h**) in UC. (**i**) Phenotypic description of the Ki67^+^ cluster in the CD8^+^ T-cell t-SNE map of melanoma and (**j**) of UC. Histograms depict expression profile of functional markers in the Ki67^+^ CD8^+^ cluster (orange) compared to total CD8^+^ T-cell population (grey). (**k**) Lineplot with fold induction of Ki67^+^ cells in CD8^+^ T-cell t-SNE map of non-responders (NR) and responders (R) to anti-PD-1 at indicated times in melanoma and (**l**) in UC. Data shown are relative to pre-treatment samples. Error bar denotes ± SEM. *p* value calculated using two-sided Wilcoxon matched-pairs test. * *p* < 0.05, ** *p* < 0.01. (**m**) Lineplots with ratio of concentrations of serum Kyn and Trp (×100) and concentration of CXCL10 at indicated times in melanoma and (**n**) in UC. *p* value calculated using two-sided Wilcoxon matched-pairs test. ** *p* < 0.01. Wk, week; NS, not significant, TB, tumor burden.

While a significant increase in the Ki67 expressing CD8^+^ T-cell population was observed, the increases in Ki67^+^ CD8^+^ T-cell subsets co-expressing the checkpoint molecules PD-L1 or CTLA-4 were even more pronounced (Figure 3a,b). In both tumor types, Ki67^+^ CD8^+^ T-cells seemed to peak after 1 or 2 cycles of anti-PD-1 therapy (Figure 3c,d). Interestingly, in the melanoma cohort the percentage of Ki67^+^ CD8^+^ T-cells, Ki67^+^ PD-L1^+^ CD8^+^ T-cells and especially Ki67^+^ PD-1^+^ CD8^+^ T-cells at baseline and for the former two also at week 1 were positively correlated with PFS (Figure 3e). In UC, PFS correlated with the increase of Ki67^+^ CD8^+^ T-cells to tumor burden and with the increase of Ki67^+^ PD-L1^+^ CD8^+^ T-cells to tumor burden (Figure 3f). In melanoma the increase of Ki67^+^(PD-L1^+^) CD8^+^ T-cells to tumor burden did not correlate with PFS.

In a subset of 7 melanoma patients with an additional blood sample collected during anti-PD-1 treatment at a median time interval of 6 months (range: 3–16 months) after start of treatment. Ki67^+^ CD8^+^ T-cells co-expressing PD-L1 or CTLA-4 had returned to baseline levels.

Global high-dimensional mapping of flow cytometry data via the t-SNE algorithm provided more insights into this proliferating subset of CD8^+^ T-lymphocytes. t-SNE analysis revealed a highly Ki67-positive CD8^+^ T-cell cluster, already present before treatment in melanoma and UC (Figure 3g,h). Compared to the total CD8^+^ T-cell population this Ki67^+^ CD8^+^ T-cell cluster demonstrated enriched expression of the T-cell activation marker HLA-DR and the immune checkpoint molecule IDO1 (Figure 3i,j). A variable expression for CTLA-4, PD-1 and its ligand PD-L1 was present in this cluster, with cells either expressing or lacking these markers. To assess the dynamics of this cluster during therapy, we manually gated on this cluster in the individual t-SNE map of each patient on each time point. Independent of response to immunotherapy, the frequency of Ki67^+^ CD8^+^ T-cells increased at week 1 and this was maintained at week 6 in melanoma patients (Figure 3k). In UC patients, the increase in Ki67 expressing CD8^+^ T-cells tended to be higher in responders (Figure 3l).

In addition, a significant increase in serum CXCL10 and Kyn/Trp was observed after 1 cycle of anti-PD-1 in the melanoma cohort (Figure 3m). The magnitude of these increases was not significantly different between responders and non-responders. The increases in CXCL10 and Kyn/Trp were not significant in the UC cohort (Figure 3n).

To conclude, via a manual gating approach and an unsupervised clustering approach we report marked invigoration of CD8^+^ T-cell subsets that have enriched expression of the activation marker HLA-DR and variably express immune checkpoint molecules. upon anti-PD-1 treatment initiation These proliferating CD8^+^ T-cell populations peaked after 1 to 2 cycles of anti-PD-1 in both melanoma and UC patients. Altogether these data point to the possible clinical significance of baseline Ki67^+^ CD8^+^ T-cells and mainly the PD-1 expressing subset in melanoma. In UC the early increase of Ki67^+^ CD8^+^ T-cells and of the PD-L1 expressing subset relative to tumor burden seems to be crucial for PFS.

### 3.4. Systemic Immune Changes after SBRT

To explore the impact of SBRT, the dynamics of immune cell frequencies before and after SBRT were investigated (resp. blood samples collected at week 1 and 2 in melanoma and week 5 and 6 in UC arm B). As described above, proliferation of the T-cell compartment peaked at the first on-treatment blood sample, which was collected before SBRT administration. No additional increases in (Ki67 expressing) T-cell subsets were detected after SBRT in melanoma nor in UC. In melanoma, modest increases in the serum concentration of CXCL10 and Kyn/Trp were observed, while the frequency of B-cells decreased (Figure S2a), but these changes were not significantly different to the observed trend before SBRT. In the UC cohort, these changes could not be confirmed (Figure S2b).

### 3.5. FlowSOM Analysis to Discover Immune Signatures Correlating with Clinical Outcome

**Figure 4:**
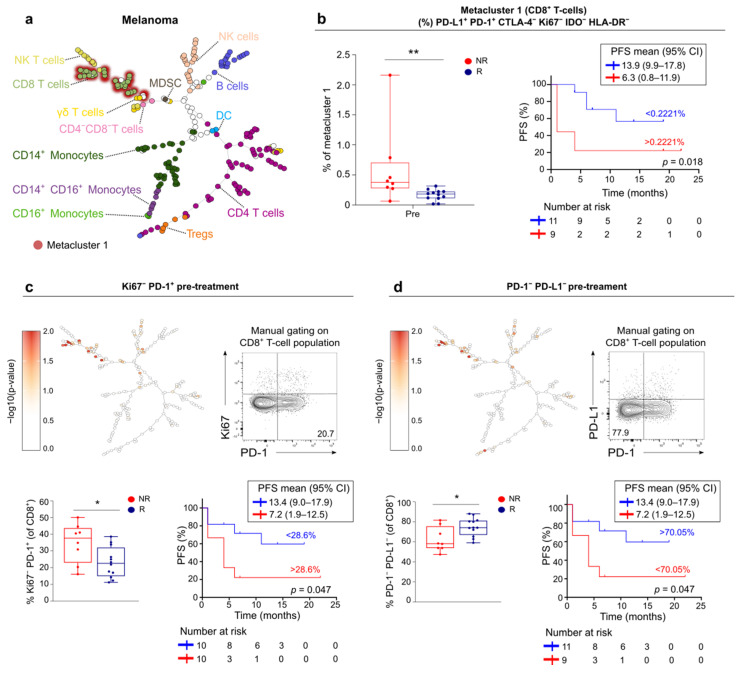
Pre-treatment expression of PD-1 and PD-L1 in non-proliferating CD8^+^ T-cells correlates with non-response to anti-PD-1 in melanoma. (**a**) FlowSOM tree of concatenated flow cytometry data of PBMCs from 20 melanoma patients (85 samples). (**b**) (left) Boxplot of the pre-treatment expression of indicated signature in metacluster 1 (CD8^+^ T-cells) in non-responders (NR) and responders (R). *p* value calculated using two-sided Mann-Whitney U test. ** *p* < 0.01. (right) Kaplan-Maier estimate of PFS stratified according to low (<0.2221%) or high (>0.2221%) expression of PD-L1^+^ PD-1^+^ CTLA-4^−^ Ki67^−^ IDO^−^ HLA-DR^−^ in metacluster 1. *p* value calculated using log-rank test. (**c**,**d**) (top left) Melanoma FlowSOM tree depicting differences in expression of the indicated signature in clusters between non-responders and responders at pre-treatment. −log10(*p* values) are plotted on FlowSOM tree showing the significantly over- or underrepresented clusters in non-responders versus responders. (top right) Contour plot (*n* = 10) representing manual gating strategy on total CD8^+^ T-cell population to confirm FlowSOM signature. (below left) Boxplots with expression of manually gated signature in non-responders (NR) and responders (R), *p* value in boxplots calculated using two-sided Mann-Whitney U test. * 0.01 < *p* < 0.05. (below right) Kaplan-Maier estimate of PFS stratified according to low or high expression of indicated signature. *p* value calculated using log-rank test. (**b**–**d**). Whiskers of boxplots extend to the minimum and maximum data point, with the horizontal line indicating the median.

In order to detect discrete differences in the systemic immune response between responders and non-responders, we applied the algorithm FlowSOM to the flow cytometry dataset. FlowSOM, a powerful clustering-based technique to explore cellular heterogeneity, generates a Minimum Spanning Tree with each node existing of a group of phenotypically related cells [19]. The 85 fcs files of melanoma patients were concatenated into one single FlowSOM tree for all individuals (Figure 4a). The frequency of cells assigned to a specific metacluster or cluster were compared between responders and non-responders before and during treatment. No differences between responders and non-responders in the percentage of cells assigned to a specific metacluster or cluster were noticed. We further explored differences between melanoma responders and non-responders by investigating the extent of (co-) expression of 6 functional markers (CTLA-4, Ki67, IDO, PD-1, PD-L1 and HLA-DR) in the FlowSOM clusters. Features distinguishing responders and non-responders were predominantly found in the T-cell compartment.

We first focused on features in (meta-) clusters corresponding to CD8^+^ T-cells. FlowSOM assigned CD8^+^ T-cells to 1 single metacluster (metacluster 1). Within this metacluster, the baseline expression of PD-L1^+^ PD-1^+^ CTLA-4^−^ Ki67^−^ IDO^−^ HLA-DR^−^ was higher in non-responding patients (Figure 4b). The frequency of CD8^+^ T-cells with this phenotype was associated with worse PFS (Figure 4b, Log-Rank analysis, *p* = 0.018). Further, multiple CD8^+^ T-cell clusters showed differential expression of Ki67^−^ PD-1^+^ and PD-1^−^ PD-L1^−^ between responders and non-responders (Figure 4c,d). We manually gated on Ki67^−^ PD-1^+^ CD8^+^ T-cells, which confirmed higher frequencies in non-responders. In contrast, manual gating on PD-1^−^ PD-L1^−^ showed decreased expression in non-responders compared to responders. These signatures were inversely correlated with each other (Spearman’s CC: −0.965, *p* < 0.001) and were both associated with PFS (Figure 4c,d).

**Figure 5:**
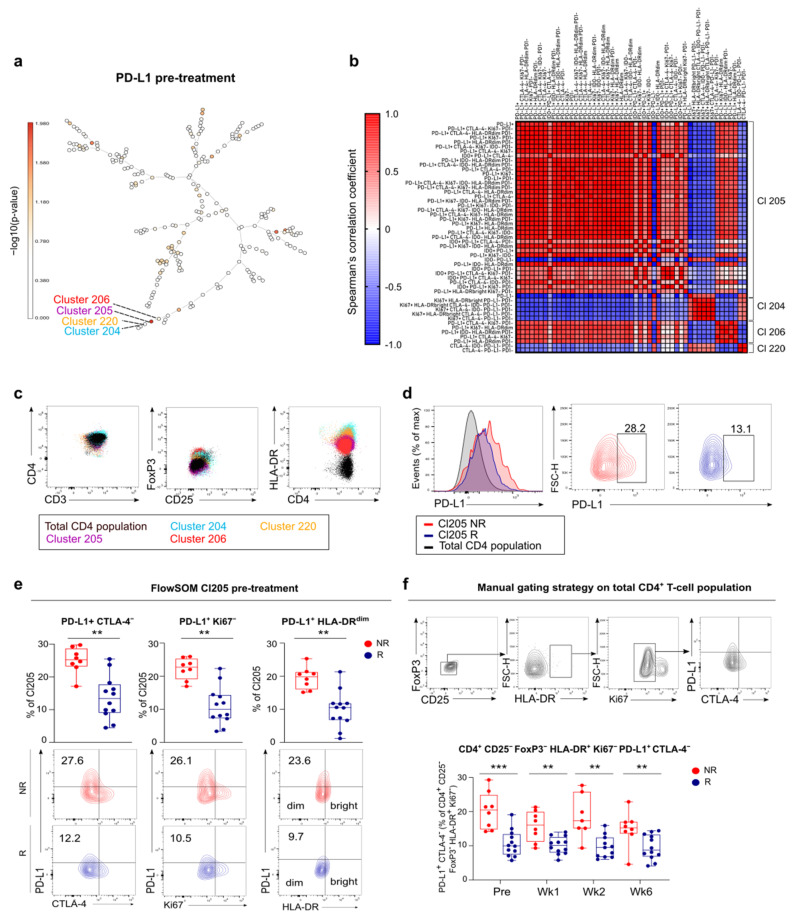
Pre-treatment expression of PD-L1 in non-proliferating CD4^+^ T-cells correlate with non-response to anti-PD-1 in melanoma patients. (**a**) Melanoma FlowSOM tree representing differences in PD-L1 expression in clusters between non-responders and responders at pre-treatment. –log10(*p*-values) are plotted on tree showing the significantly over- or underrepresented clusters in non-responders versus responders. (**b**) Correlation matrix of pre-treatment signatures (co-) expressing PD-L1 in FlowSOM clusters corresponding to CD4^+^ T-cells. Colored boxes represent Spearman’s correlation with a significance of *p* < 0.05. Red to blue represents correlation coefficients ranging from 1 to -1, respectively. (**c**) Representative flow plots of 10 independent melanoma patients with the phenotype of indicated clusters. (**d**) Histogram and contour plots with PD-L1 expression in cluster 205 of non-responders (NR, *n* = 5) versus cluster 205 of responders (R, *n* = 5) versus the total CD4^+^ T-cell population (*n* = 10). (**e**) (top) Boxplots with the frequency of expression of PD-L1 combined with CTLA-4, Ki67 or HLA-DR in cluster 205 in non-responders (NR) and responders (R). (bottom) Contour plots with indicated signatures in cluster 205 in non-responders (NR, *n* = 5) and responders (R, *n* = 5). (**f**) (top) Contour plots representing manual gating strategy of PD-L1^+^ CTLA-4^−^ Ki67^−^ HLA-DR^+^ CD4^+^ T-cells. (bottom) Boxplots with frequency of manually gated signature in CD4^+^ T-cell population at indicated times. (**e**,**f**). Whiskers of boxplots extend to the minimum and maximum data point, with the horizontal line indicating the median. *p* value calculated using two-sided Mann-Whitney U test. ** *p* < 0.01, *** *p* < 0.001. Wk, week.

In the CD4^+^ T-cell compartment, 51 signatures were detected to be differentially expressed pre-treatment between responders and non-responders in melanoma (cluster 204, cluster 205, cluster 206 and cluster 220). Notably, all signatures involved PD-L1 expression and were highly interrelated (Figure 5a,b). The majority of signatures distinguishing responders from non-responders were expressed in cluster 205, which is a HLA-DR positive CD25^−^ FoxP3^−^ CD4^+^ T-cell population (Figure 5c). Non-responders had an increased expression of PD-L1 in this cluster compared to responders (*p* = 0.0041, Figure 5d). PD-L1^+^ CD4^+^ cells in cluster 205 of non-responders were negative for expression of CTLA-4 or Ki67 and were HLA-DR^dim^ (Figure 5e). This phenotype of CD4^+^ T-cells could be confirmed via a manual gating approach. Non-responders did not only have a higher frequency of this subset of CD4^+^ T-cells at baseline but also during treatment (Figure 5f).

Since FlowSOM assigned Tregs to the same metacluster as other CD4^+^ T-cell populations in melanoma, Treg clusters were analyzed separately by defining them as one metacluster. Co-expression patterns in this Treg metacluster (including cluster 207, cluster 208, cluster 221, cluster 222 and cluster 223) were investigated. Non-responders were found to have less Tregs with phenotype HLA-DR^+^ PD-L1^−^ IDO^−^ during treatment (Figure S3a). This was confirmed via a manual gating approach (Figure S3b).

**Figure 6:**
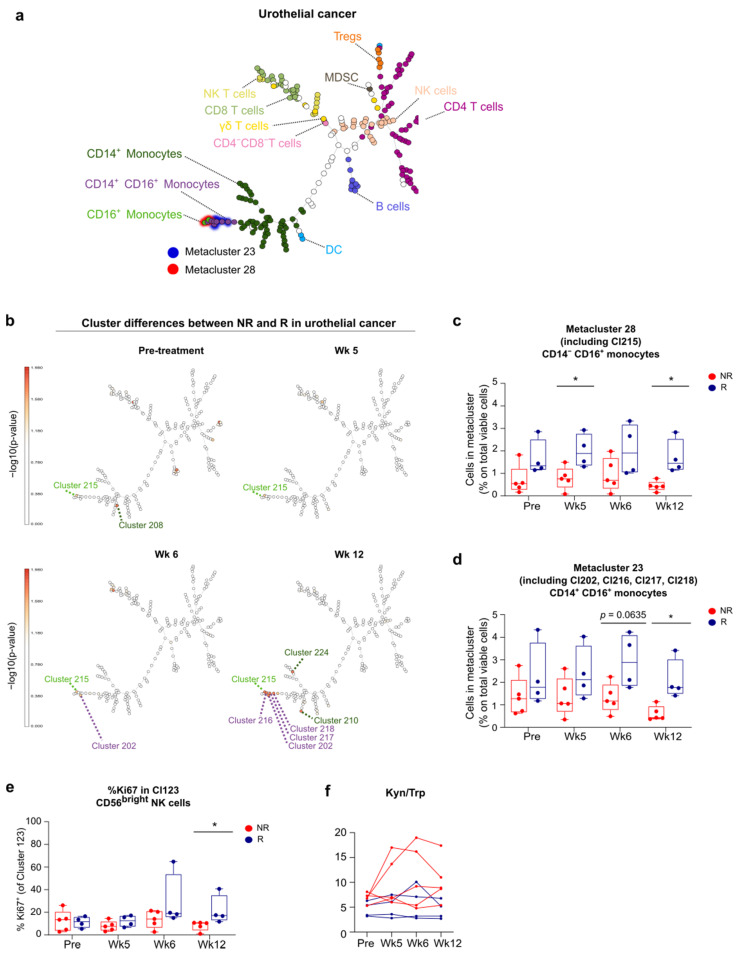
Increased frequency of monocytes associates to response in urothelial cancer. (**a**) FlowSOM tree of concatenated flow cytometry data of PBMCs from 9 UC patients (arm B, 36 samples). (**b**) UC FlowSOM trees depicting differences in the percentage of cells assigned to clusters between non-responders and responders at pre-treatment, week 5, week 6 and week 12 of anti-PD-1 treatment. −log10(*p* values) are plotted on trees showing the significantly over- or underrepresented clusters in non-responders versus responders. Colors of cluster numbers correspond with immune cell populations in a. (**c**) Boxplots with percentages of metacluster 28 corresponding to CD14^−^ CD16^+^ monocytes in non-responders (NR) and responders (R) at indicated times. (**d**) Boxplots with percentages of metacluster 23 corresponding to CD14^+^ CD16^+^ monocytes in non-responders (NR) and responders (R) at indicated times. (**e**) Boxplots with percentages of Ki67 expression in cluster 123 corresponding to CD56^bright^ NK cells in non-responders (NR) and responders (R) at indicated times. (**f**) Lineplots with the ratio of concentrations of serum Kyn and Trp (×100) in non-responders (NR) and responders (R) at indicated times. (**c**–**e**) Whiskers of boxplots extend to the minimum and maximum data point, with the horizontal line indicating the median. *p* value calculated using two-sided Mann-Whitney U test. * 0.01 < *p* < 0.05.

A similar strategy was applied to the UC cohort, concatenating 35 fcs files of the 9 arm B patients into one single FlowSOM tree (Figure 6a). Other than in the melanoma cohort, analysis in the UC cohort predominantly revealed alterations in (meta-) clusters corresponding to monocytes. Cluster 215 containing non-classical CD14^−^ CD16^+^ monocytes was overrepresented in responders before and during treatment (Figure 6b). The frequency of cells in metacluster 28, which includes cluster 215, was different between responders and non-responders at week 5 and week 12 (Figure 6c). Cluster 202, cluster 216, cluster 217 and cluster 218 contain CD14^+^ CD16^+^ monocytes and were overrepresented in responders at week 12 (metacluster 23, Figure 6d). Baseline metacluster 23 and metacluster 28 were both inversely correlated with baseline tumor burden (resp. Spearman’s CC: −0.817, *p* = 0.007 and Spearman’s CC: −0.833, *p* = 0.005), and also inversely correlated with the serum Kyn/Trp ratio (resp. Spearman’s CC: −0.817, *p* = 0.007 and Spearman’s CC: −0.900, *p* = 0.001). Furthermore, 3 additional clusters with classical CD14^+^ CD16^−^ monocytes were overrepresented in responders (cluster 208 before treatment *p* = 0.0159, cluster 210 and cluster 224 at week 12, both *p* = 0.0159), although not reflected on metacluster level (Figure 6b). In addition enhanced expression of proliferation marker Ki67 in cluster 123 (corresponding to CD56^bright^ NK cells) at week 12 was found to be associated with lower Kyn/Trp ratio and better response (Figure 6e,f).

Altogether, the results obtained by FlowSOM analysis highlight distinct signatures in melanoma and UC that correlate with clinical outcome. In melanoma, these signatures were predominantly found in the lymphoid compartment and mainly involved different baseline expression patterns of PD-1 and/or PD-L1: the expression of PD-L1/PD-1 in non-proliferating (Ki67^−^) CD8^+^ and CD4^+^ T cells was associated with worse clinical outcome. In the UC cohort signatures with a higher frequency of (non-) classical monocytes were found to be correlated with response, but also had a strong inverse correlation with tumor burden.

### 3.6. Link between Blood and Tumor Micro-Environment

We explored possible associations between the systemic immune landscape and the TILs score/PD-L1 expression in the tumor. In melanoma, patients with a high TILs score (score 3 versus score 1 and 2) had a significantly lower frequency of circulating PD-L1^+^ CD4^+^ T-cells and PD-L1^+^ PD-1^+^ CD4^+^ T-cells (Figure S4a). This could not be confirmed in the UC cohort (Figure S4b). No correlation between PD-L1 staining in the tumor micro-environment and systemic immune features could be observed for both cohorts.

## 4. Discussion

In this study we conducted an in-depth analysis of baseline and on-treatment systemic immune features in a cohort of melanoma and UC patients treated with anti-PD-1 therapy combined with SBRT in a similar design.

Baseline immunity (before start of treatment) was clearly different between these two cohorts, supporting a less active immune landscape in UC compared to melanoma. NLR was significantly higher in the UC cohort. Variations in baseline NLR have been reported between tumor types and increased pre-treatment NLR has been linked to worse outcome in patients treated with immunotherapy [20]. The NLR is considered as a marker reflecting the balance between inflammation state (pro-tumoral) and adaptive immune surveillance and response (anti-tumoral). UC patients also had higher frequencies of classical monocytes and immunosuppressive MDSCs. In the melanoma cohort, cells of the lymphoid lineage were higher as reflected by higher frequencies of lymphocytes in total, γδ T-cells and proliferating B-cells. In line with this, higher serum concentrations of CXCL10, an IFNγ-inducible chemokine involved in T-cell recruitment to the tumor [21,22], were measured in melanoma compared to UC. In contrast, serum concentrations of soluble MICA and MICB were higher in UC patients. MICA and MICB are ligands for the activating receptor NKG2D and their soluble form has been implicated in the perturbation of effector immune cell function and the stimulation of MDSCs [23].

The observation of a distinct baseline systemic immunity in the 2 cohorts may play a prominent role in the different response rates to immunotherapy. The objective response rate (ORR) of anti-PD-1 monotherapy reported in inoperable stage III/IV melanoma is around 42–45%, while ORR reported in chemotherapy-refractory metastatic urothelial cancer is considerably lower (15–28.6%) [24,25,26,27]. Currently, our understanding of intrinsic factors such as tumor type and burden, patient age and sex, and extrinsic factors such as prior systemic treatments that shape the immune system is far from complete. Tumor mutational burden has been linked to response to immunotherapy and varies across tumor types, with melanoma constituting the largest neoantigen repertoire [8,28]. Both patients’ age and sex were evidenced to impact the driver mutations that arise during tumorigenesis, with younger and female patients accumulating driver mutations that are less readily presented by MHC molecules [29]. In contrast, in a meta-analysis including more than 10,000 patients treated with immunotherapy for several types of advanced cancers, a higher relative reduction of the risk of death was observed in male compared to female patients [30]. Since a higher tumor mutational burden has been reported in male patients [31,32] and this is a predictor of benefit from immune checkpoint inhibitors [33,34], this could be a possible explanation for improved overall survival rates in male patients. Aging has been reported to accompany certain immune changes such as a decrease in the number and functionality of naïve CD8^+^ T-cells [35,36] and reduced phagocytic function and HLA-II expression of DCs [37], indicating elder individuals have an impaired T-cell response to cross-presented antigens (immunosenescence). Nevertheless, a large multi-centric study reported that older melanoma patients had better response to anti-PD-1 treatment compared to younger patients [38]. In our study median age in the UC cohort was higher in responders compared to non-responders (75.5 versus 61.0 years). Age was not correlated with NLR in the melanoma nor the UC cohort, which is consistent with other reports [39,40]. The depicted reference values of neutrophils and lymphocytes (Figure 2a) further support baseline differences per tumor type independent of sex and age.

Importantly, the majority of UC patients received prior chemotherapy and one third even received two or three treatment lines before trial inclusion, which may have altered the immune landscape considerably. The impact of these immunological alterations on immunotherapy response is unclear. A number of recent studies hypothesize that chemotherapy may sensitize tumors for immunotherapy whereas others postulate that chemotherapy negatively impacts myelopoiesis, induces inflammation and increased expression of immunosuppressive molecules such as IDO and PD-L1 [41,42,43,44,45].

The observations in this study demonstrate important differences in baseline immunity between and within tumor types and these may be important determinants for immunotherapy response. Better insights into the various intrinsic and extrinsic factors that shape this baseline immunity may be relevant in order to gain further insights how to optimize immunotherapy response across various cancer types.

Pathological response predictive for clinical outcome to immunotherapy has been reported early after initiation of anti-PD-1 in melanoma [34,46] and the accumulation of CD8^+^ T-cells expressing inhibitory receptors (exhausted T-cells, T_ex_) was detected in the peripheral blood within 3 weeks after immunotherapy initiation [46,47,48]. In the current study, we observed increased proliferation of CD8^+^ T-cells in the blood as early as 7 days after anti-PD-1 treatment initiation in melanoma patients. Similar increases in Ki67^+^ CD8^+^ T-cells were detected after one or two treatment cycles in UC patients. Proliferating CD8^+^ T-cells were positive for the activation marker HLA-DR and for IDO and had variable expression of checkpoint molecules such as PD-1, PD-L1 and CTLA-4.These findings are in line with previous data in NSCLC and melanoma, where anti-PD-1 was reported to revitalize an already existing T-cell response consisting of primed (tumor-specific) CD8^+^ T-cells that had become exhausted due to chronic antigen stimulation [46,47,48]. It has been hypothesized by Huang et al. that reinvigoration of T_ex_ occurs in the peripheral blood prior to migrating into the tumor as supported by a single peak of PD-1-blockade-induced immune reinvigoration despite on-going treatment [46,47]. In line with this, proliferating CD8^+^ T-cells in the current study peaked early in the PBMC compartment and declined upon further anti-PD-1 administration.

No clear immune boost effect could be observed after SBRT in these 2 small patient cohorts except from a moderate increase in CXCL10 in the melanoma cohort.

In melanoma, proliferation of the total CD8^+^ T-cell population, PD-L1^+^ CD8^+^ T-cells and PD-1^+^ CD8^+^ T-cells at baseline were correlated with prolonged PFS. The former two populations were also correlated with PFS after one cycle of anti-PD-1 (PD-1 expression was not measurable beyond baseline presumably due to anti-PD-1 treatment preventing the in vitro added PD-1 antibodies from binding their epitopes). In contrast, FlowSOM analysis supports a negative impact of baseline PD-1/PD-L1 expression in non-proliferating (Ki67^−^) T-helper (CD25^−^ Foxp3^−^ CD4^+^) and cytotoxic T-cells (CD8^+^). A negative prognostic effect of PD-L1 expressing CD8^+^ T-cells in melanoma has been reported in the context of anti-CTLA-4 immunotherapy and also in early stage melanoma without systemic treatment [49,50]. FlowSOM analysis also revealed PD-1/PD-L1 co-expression on circulating CD8^+^ T-cells. This has been described before, and PD-1 and PD-L1 were shown to bind in cis with high affinity in in vitro lentivirally transduced cell cultures, including Jurkat Cells, evidencing this interaction can also occur on T-cells in vivo [51]. These in cis PD-1/PD-L1 interactions on CD8^+^ T-cells might reflect functional inactivation, which would explain the enhanced co-expression of PD-1 and PD-L1 on CD8^+^ T-cells in non-responders observed in this study. In addition PD-L1/PD-1 co-expressing CD4^+^ T-cells in blood tend to be related to a lower TILs score at the level of tumor micro-environment in our melanoma cohort. In UC patients, the expansion of proliferating (Ki67^+^) CD8^+^ T-cells and its PD-L1^+^ subset relative to tumor burden was correlated with longer PFS.

These data support that the size of the proliferating cytotoxic T-cell compartment and its expansion is closely involved in the immunotherapy response. As UC patients have lower baseline lymphocyte counts compared to melanoma, the actual magnitude of the expansion might be important for response initiation. In addition, in arm B of the UC cohort tumor burden was significantly lower in responders versus non-responders, which may explain why the ratio is of importance in the UC cohort. Huang et al. have reported that the magnitude of the reinvigoration of T_ex_ as a ratio to pre-treatment tumor burden was correlated with clinical outcome in immunotherapy in melanoma [47]. The fact that tumor burden in arm B of the UC cohort was significantly lower in responders compared to non-responders, may be a reason why this ratio was related to response only in the UC cohort in this study. Our data are also supported by data from the neo-adjuvant setting where a single injection of pembrolizumab in resectable stage III or IV melanoma patients resulted in the expansion of Ki67^+^ PD-1^+^ CTLA-4^+^ CD8^+^ T-cells in the peripheral blood of patients 7 days post injection. This Ki67^+^ CD8^+^ T-cell population was demonstrated to be present in the blood before start of the treatment and supports the reinvigorating properties of anti-PD-1 therapy on a preexisting immune response [52]. In the study of Huang et al. the CD8^+^ T-cell population responding to anti-PD-1 treatment was characterized as CD45^lo^ CD27^hi^, containing cells with high expression of CTLA-4, 2B4 and PD-1. Moreover this population was Eomes^hi^ and T-bet^lo^, which is consistent with an exhausted T-cell phenotype [47]. Although the proliferating CD8^+^ T-cells in our study had higher expression of the activation marker HLA-DR compared to the non-proliferating CD8^+^ T-cells, they also had higher IDO expression and variable expression of PD-1 and PD-L1. The expression of these immune checkpoint molecules has been shown to be a possible physiological negative feedback mechanism upon immune stimulation [45,53]. These data may explain conflicting results on the prognostic value of checkpoint molecules expressed on immune cells.

These data also underline the relevance of analyzing PD-1/PD-L1 expression on circulating T-cell subsets. Whereas PD-1 is predominantly expressed on lymphocytes, its ligand PD-L1 has been detected on a variety of cells in the tumor microenvironment including conventional DCs, macrophages, MDSCs, and extracellular vesicles [54,55,56,57]. Blockade of PD-L1 signaling on immune cells (especially DCs and macrophages) was demonstrated to be critical for an optimal anti-tumor immune response, as opposed to/in addition to cancer-cell intrinsic PD-L1 expression [55,56]. This may explain the inconsistent observations on the role of tumor PD-L1 expression in predicting response to PD-1 blockade, and why its absence does not preclude response [58]. Although PD-L1 expression in tumor tissue has been related to response to PD-1 blockade, a systematic evaluation of 45 FDA-approved trials involving 15 tumor types demonstrated that PD-L1 expression was predictive in only 28.9% of cases [6]. PD-L1 expression on circulating T-cells is less studied. Pre-treatment PD-L1 expression on peripheral CD8^+^ and CD4^+^ T-cells was associated with worse outcome in melanoma patients receiving CTLA-4 blockade [49]. We previously reported that the frequency of circulating PD-L1^+^ CD8^+^ T-cells in early-stage melanoma was an independent prognostic marker. High frequencies of PD-L1^+^ CD8^+^ T-cells were associated with other immune suppressive features including increased Kyn/Trp ratio (implying increased IDO1 activity) and increased MDSCs and Tregs [50]. Together with the observation in the current study that the level of PD-L1 on circulating CD4^+^ and CD8^+^ T-cells is of importance for the outcome of anti-PD-1 treatment, these findings suggest that PD-L1 expression in the lymphocyte compartment might be an important blood biomarker in cancer patients receiving PD-1 blockade.

FlowSom analysis in the UC cohort revealed higher frequencies of monocytes in responding UC patients. High frequencies of non-classical CD14^−^ CD16^+^ monocytes and intermediate CD14^+^ CD16^+^ monocytes were closely correlated with lower tumor burden at baseline. The percentage of proliferating CD56^bright^ NK cells was also found to be increased in responding UC patients at week 12. Intratumoral CD56^bright^ NK cells have been previously reported to be associated with improved survival outcomes in localized stage bladder cancer [59]. At week 12 responding UC patients also had lower levels of Kyn/Trp, suggesting decreased activity of IDO1, an enzyme that is implicated in acquired immune tolerance [57,60].

The immunotherapy field in oncology is rapidly changing with superior long-term results of combination immunotherapy in melanoma and renal cell carcinoma [61] and very promising results in melanoma in the neoadjuvant setting that seem to be extendable to other tumor types [62,63]. Moreover the number of clinical trials with new immune targets is increasing e.g., TIM-3, LAG-3, GITR, TIGIT. Immune monitoring of peripheral blood is attractive for dynamic monitoring of the immune system, which ideally could lead to a strategy of treatment adaptation in order to optimize response. In the current study blood signatures before and during treatment with anti-PD-1 therapy combined with SBRT were investigated. Whether the observed signatures related to clinical outcome are applicable in daily practice and can be extrapolated to other immunotherapy regimens such as the combination of anti-PD-1 with anti-CTLA4 needs to be further investigated. Distinct cellular mechanisms of anti-PD-1 or anti-CTLA-4 monotherapy compared to combination therapy have been detected in the peripheral blood [64,65] and anti-CTLA4 monotherapy has been shown to induce some immune landscape changes in blood that are considered negative for response on subsequent anti-PD-1 treatment [52]. These immune monitoring data can provide relevant insights in how to optimize immunotherapy strategy.

## 5. Conclusions

Despite the limitations of small sample sizes, use of cryopreserved samples and multiple testing in the FlowSom analysis, this study clearly reveals a different baseline immune landscape in melanoma and UC which may be of importance for immunotherapy response. The intrinsic (host and/or tumor related) and extrinsic factors (e.g., prior treatments) that shape this immune landscape are currently incompletely understood. Better insights in these determinants may be important to gain new insights for optimizing immunotherapy outcome. This study also reports signatures of proliferation in the CD8^+^ T-cell compartment prior to and early after anti-PD-1 initiation that were positively correlated with clinical outcome. Moreover our data support the clinical relevance of PD-1/PD-L1 expression on circulating immune cell subsets in melanoma.

## Data Availability

Data is contained within the article or Supplementary Material.

## Supplementary Materials

The following are available online at https://www.mdpi.com/article/10.3390/cancers13112630/s1, Figure S1: Gating strategies of immune cell populations analyzed in this study, Figure S2: Systemic immune changes after SBRT, Figure S3: HLA-DR^+^ PD-L1^−^ IDO^−^ expressing regulatory T-cells are upregulated in responding melanoma patients, Figure S4: Abundance of TILs is linked with blood PD-L1 and PD-1 expression on CD4^+^ T-cells, Table S1: Patient characteristics of melanoma patients, Table S2: Patient characteristics of urothelial cancer patients, Table S3: List of monoclonal antibodies for flow cytometry.
