# Supplementary material for: Immune Monitoring in Melanoma and Urothelial Cancer Patients Treated with Anti-PD-1 Immunotherapy and SBRT Discloses Tumor Specific Immune Signatures
Source: Cancers (Basel). 2021 May 27;13(11):2630. doi: 10.3390/cancers13112630 (PMC8198315; doi:10.3390/cancers13112630)
Supplement: Supplementary file 1 [file cancers-13-02630-s001.zip › cancers-1164786-supplementary.pdf]

cancers

MDPI

Supplementary Material: Immune Monitoring in Melanoma and Urothelial Cancer Patients Treated with Anti-PD-1 Immunotherapy and SBRT Discloses Tumor Specific Immune Signatures

Annabel Meireson, Simon J. Tavernier, Sofie Van Gassen, Nora Sundahl, Annelies Demeyer, Mathieu Spaas, Vibeke Kruse, Liesbeth Ferdinande, Jo Van Dorpe, Benjamin Hennart, Delphine Allorge, Filomeen Haerynck, Karel Decaestecker, Sylvie Rottey, Yvan Saeys, Piet Ost and Lieve Brochez

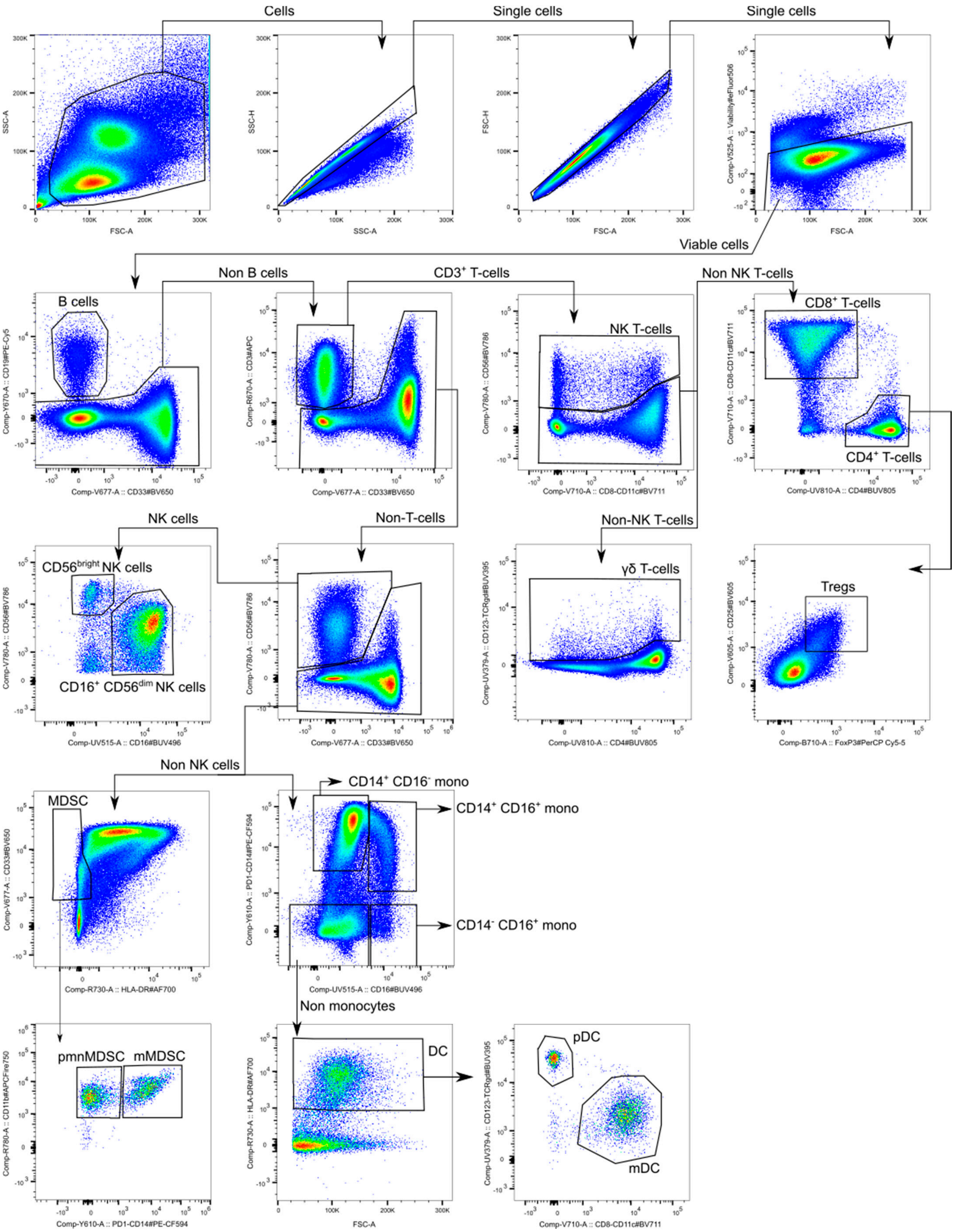

Figure S1. Gating strategies of immune cell populations analyzed in this study. Tregs, regulatory T-cells; MDSC, myeloid-derived suppressor cells; pmnMDSC, polymorphonuclear MDSC; mMDSC, monocytic MDSC; mono, monocytes; DC, dendritic cells; pDC, plasmacytoid DC; mDC, myeloid DC.

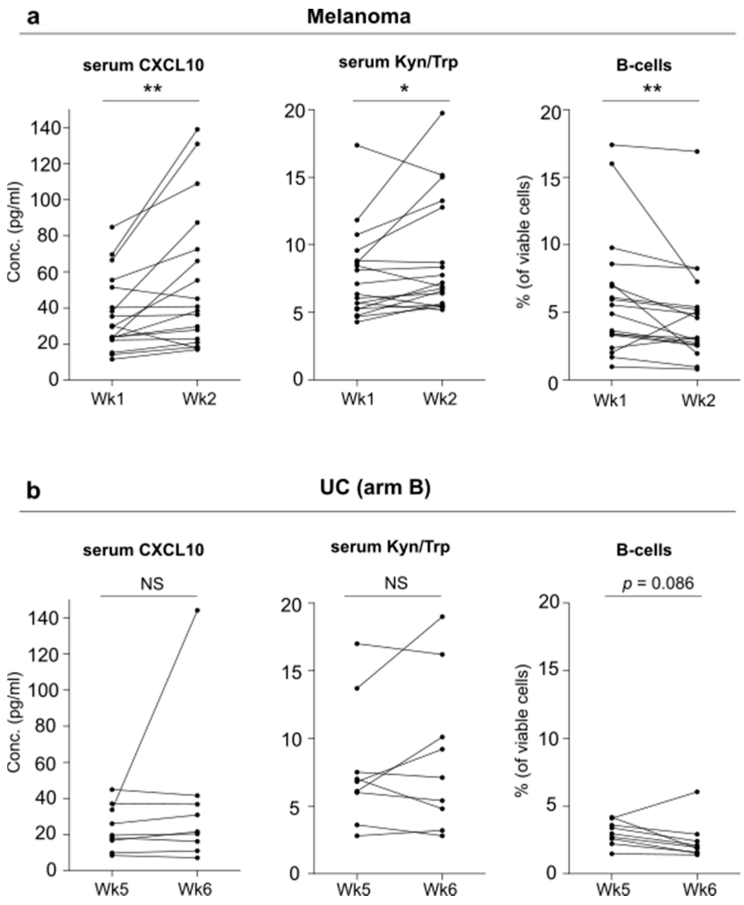

Figure S2. Systemic immune changes after SBRT. (**a**) Lineplots with the concentration of serum CXCL10, ratio of concentrations of serum Kyn and Trp (×100) and the frequency of B-cells before and after SBRT in melanoma and (**b**) in UC (arm B). *P* value calculated using Wilcoxon Signed Ranks Test. * $p < 0.05$ and ** $p < 0.01$. NS, not significant; Wk, week.

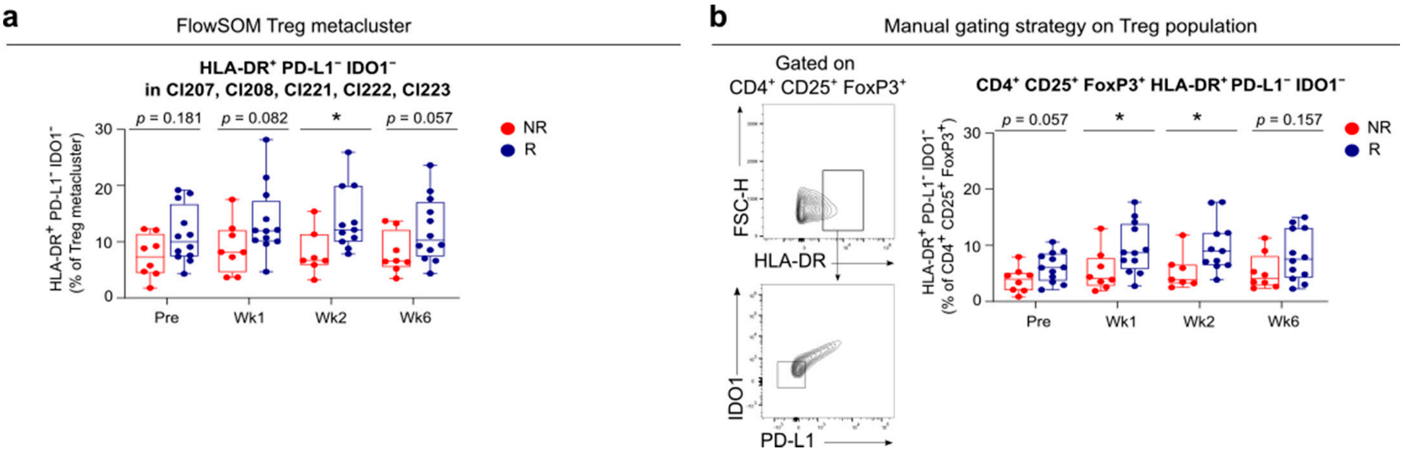

Figure S3. HLA-DR⁺ PD-L1⁻ IDO⁻ expressing regulatory T-cells are upregulated in responding melanoma patients. (**a**) Boxplots with frequency of HLA-DR⁺ PD-L1⁻ IDO⁻ expression in manually assigned Treg metacluster in non-responders (NR) and responders (R). (**b**) (left) Contour plots representing manual gating strategy of HLA-DR⁺ PD-L1⁻ IDO⁻ expression in Treg (CD4⁺ CD25⁺ FoxP3⁺) population. (right) Boxplots with frequency of manually gated HLA-DR⁺ PD-L1⁻ IDO⁻ expression in Treg population. Whiskers of boxplots extend to the minimum and maximum data point, with the horizontal line indicating the median. *p* value calculated using two-sided Mann-Whitney U test. * $p < 0.05$. Wk, week.

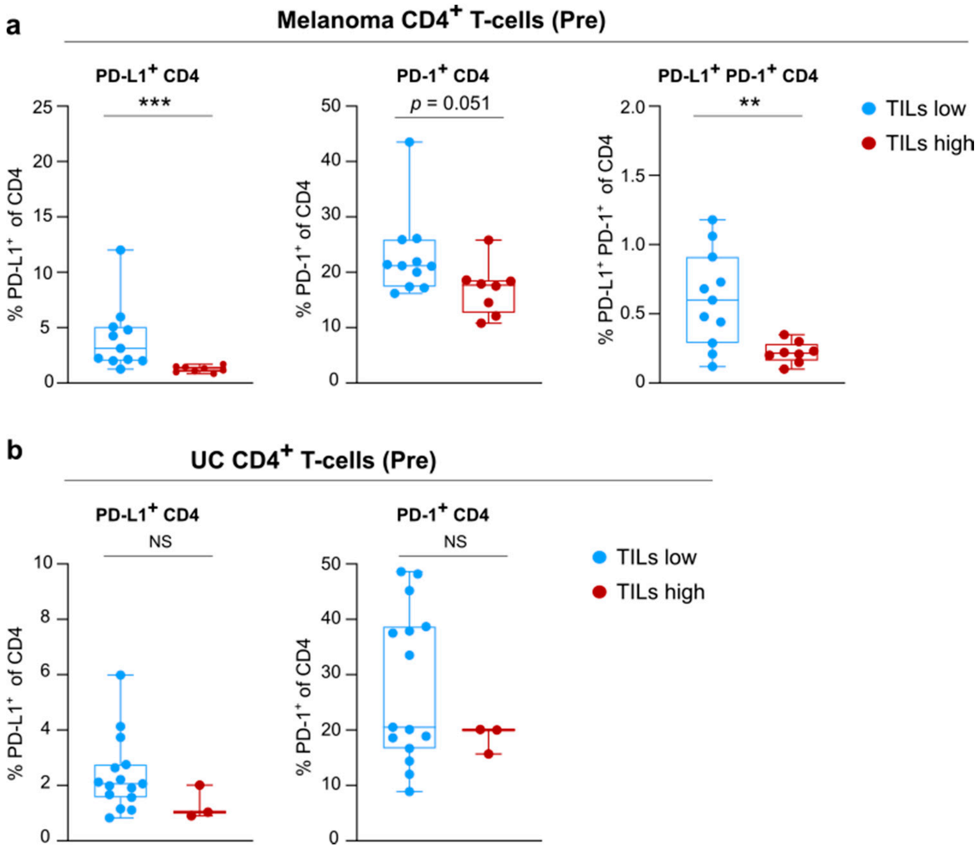

Figure S4. Abundance of TILs is linked with blood PD-L1 and PD-1 expression on CD4+ T-cells. (**a**) Boxplots with frequency of pre-treatment PD-L1+, PD-1+, PD-L1+ PD-1+ expression on CD4+ T-cells according to TILs score in tumor tissue of melanoma patients. (**b**) Boxplots with frequency of pre-treatment PD-L1+ and PD-1+ expression on CD4+ T-cells according to TILs score in tumor tissue of UC patients. Whiskers of boxplots extend to the minimum and maximum data point, with the horizontal line indicating the median. *P* value calculated using two-sided Mann-Whitney U test. ** $p < 0.01$ and *** $p < 0.001$. NS, not significant; Pre, pre-treatment; Wk, week. TILs were evaluated semi quantitatively: 1+, sporadic TILs; 2+, moderate number of TILs; 3+, abundant occurrence of TILs. For dichotomization, the TILs score was categorized into 'low' (1+ or 2+) and 'high' (3+).

Table S1. Patient characteristics of melanoma patients.

Characteristic	Melanoma (*n* = 20)
Age, median (range), y	60.5 (34.0–78.0)
Male sex, *n* (%)	13 (65)
Karnofsky PS, *n* (%)	
90-100	17 (85)
70-80	3 (15)
Primary melanoma, *n* (%)	
Cutaneous	13 (65)
Uveal	1 (5)
Mucosal	1 (5)
Acral Lentiginous	3 (15)
Unknown	2 (10)
Metastasis stage, *n* (%)	
M0	5 (25)
M1a	2 (10)
M1b	3 (15)
M1c	8 (40)
M1d	2 (10)
BRAF mutation present, *n* (%)	11 (55)
Visceral disease, *n* (%)	12 (60)
Liver metastases, *n* (%)	5 (25)
LDH > ULN, *n* (%)	7 (35)
Previous systemic treatments, *n* (%)	
0	18 (90)
1	1 (5)
2	1 (5)

Abbreviations: PS, performance status; ULN, upper limit of normal.

Table S2. Patient characteristics of urothelial cancer patients.

Characteristic	arm A (*n* = 9)	arm B (*n* = 9)
Age, median (range), y	58 (54–75)	71 (50–84)
Male sex, *n* (%)	8 (89)	8 (89)
ECOG PS, *n* (%)		
0	4 (44)	6 (67)
1	5 (56)	3 (33)
Visceral disease, *n* (%)	5 (56)	6 (67)
Liver metastases, *n* (%)	2 (22)	1 (11)
Previous systemic treatments, *n* (%)		
0	2 (22)	3 (33)
≥1	7 (78)	6 (67)
≥2	3 (33)	1 (11)
3	2 (22)	0

Abbreviations: PS, performance status.

Table S3. List of monoclonal antibodies for flow cytometry.

Antigen	Fluorochrome	Company	Reference	Clone
CD123	BUV395	BD	564195	7G3
TCRγδ	BUV395	BD	564155	B1
CD16	BUV496	BD	564654	3G8
CD39	BUV737	BD	564726	TU66
CD4	BUV805	BD	564910	SK3
CD152 (CTLA-4) *	BV421	BD	565931	BNI3
LD	eFluor506	eBioscience	65-0866-14	N/A
CD25	BV605	BD	562661	2A3
CD33	BV650	BD	740573	WM-53
CD8	BV711	BD	563676	RPA-T8
CD11c	BV711	BD	563130	B-ly6
CD56	BV785	Biolegend	362549	5.1H11
Ki67 *	AF488	Biolegend	350507	Ki-67
FoxP3 *	PerCP-Cy5-5	BD	561493	236A/E7
IDO *	PE	R&D	MAB6030	#700838
CD14	PE-CF594	BD	562334	MφP-9
CD279 (PD-1)	PE/Dazzle594	Biolegend	329939	EH12.2H7
CD19	PE/Cy5	BD	560993	HIB19
CD274 (PD-L1)	PE/Cy7	Biolegend	329717	29E.2A3
CD3	APC	BD	561804	HIT3α
HLA-DR	APC-R700	BD	565128	G46-6
CD11b	APC/Fire750	Biolegend	301351	ICRF44

* Intracellular markers.
